# Virtual reality in interventional radiology education: a systematic review

**DOI:** 10.1590/0100-3984.2020.0162

**Published:** 2021

**Authors:** And Yara Particelli Gelmini, Márcio Luís Duarte, André Moreira de Assis, Josias Bueno Guimarães Junior, Francisco César Carnevale

**Affiliations:** 1 Webimagem Telerradiologia, São Paulo, SP, Brazil.; 2 Escola Paulista de Medicina da Universidade Federal de São Paulo (EPM-Unifesp), São Paulo, SP, Brazil.; 3 Instituto de Radiologia do Hospital das Clínicas da Faculdade de Medicina da Universidade de São Paulo (InRad/HC-FMUSP), São Paulo, SP, Brazil.; 4 MIP Engenharia, Belo Horizonte, MG, Brazil.

**Keywords:** Radiology, interventional, Virtual reality, Education, medical, Simulation training, Radiologia intervencionista, Realidade virtual, Educação médica, Treinamento por simulação

## Abstract

The aim of this study was to compare virtual reality simulation with other methods of teaching interventional radiology. We searched multiple databases-Cochrane Library; Medline (PubMed); Embase; Trip Medical; Education Resources Information Center; Cumulative Index to Nursing and Allied Health Literature; Scientific Electronic Library Online; and Latin-American and Caribbean Health Sciences Literature-for studies comparing virtual reality simulation and other methods of teaching interventional radiology. This systematic review was performed in accordance with the criteria established by the Preferred Reporting Items for Systematic Reviews and Meta-Analyses and the Best Evidence Medical Education (BEME) Collaboration. Eligible studies were evaluated by using the quality indicators provided in the BEME Guide No. 11 and the Kirkpatrick model of training evaluation. After the eligibility and quality criteria had been applied, five randomized clinical trials were included in the review. The Kirkpatrick level of impact varied among the studies evaluated, three studies being classified as level 2B and two being classified as level 4B. Among the studies evaluated, there was a consensus that virtual reality aggregates concepts and is beneficial for the teaching of interventional radiology. Although the use of virtual reality has been shown to be effective for skill acquisition and learning in interventional radiology, there is still a lack of studies evaluating and standardizing the employment of this technology in relation to the numerous procedures that exist within the field of expertise.

## Abstract (pt)

O objetivo deste estudo foi comparar a simulação de realidade virtual com outros métodos de aprendizagem na radiologia intervencionista. Foram realizadas buscas nas bases de dados eletrônicas Cochrane Library, Medline (PubMed), Embase, Trip Medical, Education Resources Information Center, Cumulative Index to Nursing and Allied Health Literature, Scientific Electronic Library Online, e Latin-American and Caribbean Health Sciences Literature para estudos comparando a realidade virtual com outros métodos de aprendizagem na radiologia intervencionista. Esta revisão sistemática foi realizada de acordo com o Preferred Reporting Items for Systematic Reviews and Meta-Analyses e a Best Evidence Medical Education (BEME) Collaboration. Os estudos elegíveis foram avaliados usando o questionário de Buckley no BEME Guide nº 11 e o modelo de Kirkpatrick. Cinco ensaios clínicos randomizados foram incluídos nesta revisão, após a aplicação de todos os critérios de elegibilidade e qualidade. O nível de evidência educacional encontrado entre os artigos variou, sendo três estudos nível 2B e os demais nível 4B de acordo com o modelo de Kirkpatrick. Não houve divergência entre os estudos de que a realidade virtual agrega conceitos e é benéfica para o ensino da radiologia intervencionista. O uso da realidade virtual para aquisição de conhecimento e aprendizagem em radiologia intervencionista tem se mostrado eficaz, mas ainda faltam estudos que avaliem e estruturem a utilização dessa tecnologia em relação aos inúmeros procedimentos existentes no campo de atuação.

## INTRODUCTION

Learning is defined as the smallest independent structural experience that contains an objective, an activity to carry out, and an assessment^(1,2)^. Being able to acquire learning means being aware of that process as a whole, which encompasses knowledge, skill, and attitude^(3)^.

In recent decades, minimally invasive procedures have replaced many open surgical procedures, one of the key aims being to reduce surgical morbidity and mortality^(4)^. To that end, new resources are being tested in order to improve surgical skills in the current setting, in which procedures are becoming increasing invasive and complex^(4-6)^. Because instruction in catheter-based endovascular interventions has become continuous in hospitals, a structure involving mentors is needed for resident training^(7)^.

Virtual reality (VR) is a technology that aims to immerse the user in a particular location, through the perceptual deprivation of the actual environment, using computerized equipment or previously captured video to create a setting resembling aspects of the real world^(2,8)^. Simulators based on this technology mimic realistic situations and relevant scenarios, which can then be explored by various professionals^(9)^. In interventional radiology, VR has been increasingly used for improving procedural skills, being widely employed for the teaching and improvement of surgical techniques, such as angiography, angioplasty, vascular catheterization, catheter placement under fluoroscopic guidance, and stent placement, as well as for the teaching of basic procedures such as the Seldinger technique^(4,5,7,10-12)^. Currently, VR involves numerous devices and technologies, which can be adapted to work with a variety of equipment used around the world^(13)^. However, in a study published in 2019, Nesbitt et al.^(4)^ stated that a VR simulator (VRS) cannot be purchased for less than £100,000.

The handling of basic materials, such as guidewires, catheters, drains, thermal ablation equipment, and needles, typically presents a challenge at the beginning of the learning process in interventional radiology^(14)^. Therefore, seeking to enrich the medical teaching methodology, administrators have combined VR with traditional methods, thus broadly aggregating concepts to increase effectiveness as well as to improve surgical skills^(15)^. Preliminary studies comparing the combined use of VR and traditional teaching have obtained promising results regarding the employment of VR as a teaching method^(5,6,11)^.

The aim of this study was to identify, systematically evaluate, and summarize the best available scientific evidence comparing VR with various other methods of teaching interventional radiology.

## METHODS

### Study model

This systematic review was conducted in accordance with the guidelines established by the Preferred Reporting Items for Systematic Reviews and Meta-Analyses and by the Best Evidence Medical Education (BEME) Collaboration (https://www.bemecollaboration.org/). It was registered in advance via the Open Science Framework (https://osf.io/wn762). The study was deemed exempt from formal institutional review by our institutional review board because no human or animal subjects were involved.

### Search strategies

We searched the following databases: Cochrane Library; Medline (PubMed); Embase; Trip Medical; Education Resources Information Center; Cumulative Index to Nursing and Allied Health Literature; Scientific Electronic Library Online; and Latin-American and Caribbean Health Sciences Literature. As search terms, we used US National Library of Medicine Medical Subject Headings, as follows: interventional radiology; virtual reality; augmented reality; video games; computer simulation; education, medical; teaching; and simulation training. We imposed no restrictions regarding language, origin, date of publication, publication status, or population evaluated. Reference lists of the studies selected and the main reviews on the subject were also evaluated. Manual searches were also carried out in the reference lists. All searches were performed on July 29, 2020.

### Inclusion criteria

We included studies that compared VR with other methods for teaching interventional radiology. The comparator methods included the use of a pulsatile human cadaver model (PHCM), the traditional cadaver model, didactic classes, and the porcine model.

### Study selection of studies and data extraction

Eligibility was determined on the basis of the relevance of the articles or their abstracts and the relevance of the respective journals. The identification of eligible studies was carried out in two stages by two reviewers, working independently. Disagreements were resolved by consensus. In the first stage, after excluding duplicates, the reviewers evaluated titles and abstracts, thus pre-selecting potentially eligible studies. In the second stage, the full texts of those same studies were assessed in order to confirm eligibility. The selection process was performed with the Rayyan QCRI software^(16)^.

### Evaluation of methodological quality

The Cochrane Collaboration tool was applied in order to assess the risk of bias for individual studies and across studies^(17)^. Eligible studies were evaluated by using the quality indicators provided in BEME Guide No. 11^(18)^ and the Kirkpatrick model of training evaluation described in BEME Guide No. 8 by Steinert et al.^(19)^. The tools are based on instruments that cover a wide range of methodological issues in studies evaluating teaching methodology.

## RESULTS

The systematic review yielded 5,189 articles, of which 50 were found to be duplicates. After the two independent evaluators had read the titles and abstracts of the remaining 5,139 articles, using the Rayyan online platform, 51 articles were chosen for full-text reading.

**Figure 1:**
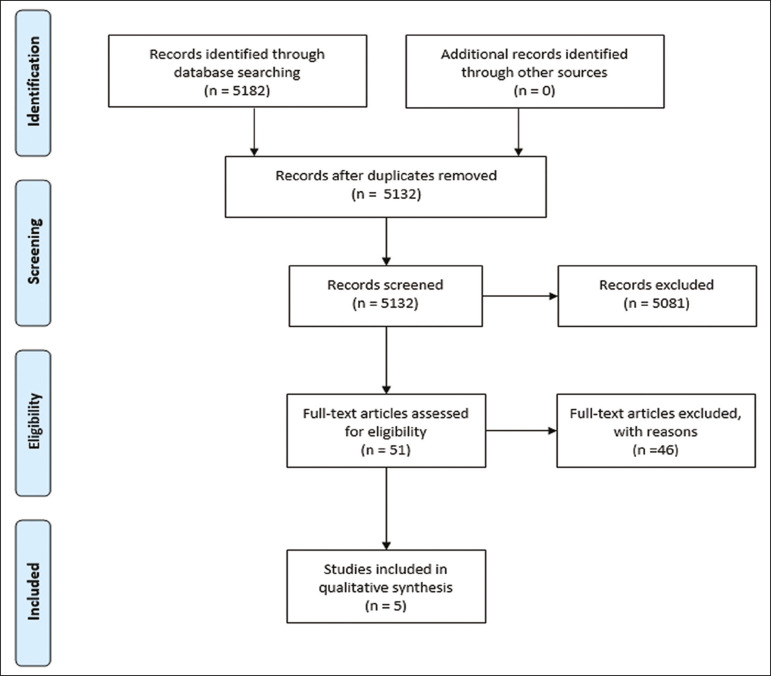
Preferred reporting items for systematic reviews and meta-analyses flow diagram of the study selection process.

Studies that did not compare teaching methods evaluated were excluded, as were those that did not employ the quality indicators provided in BEME Guide No.11^(9)^, those analyzing factors other than medical teaching, and randomized clinical trials unrelated to the field of interventional radiology. Thus, 46 studies were excluded, resulting in a final sample of five studies (Figure 1).

In the five studies selected, the samples were heterogeneous, comprising vascular surgeons; novice and experienced interventional radiologists; general surgery residents; medical students; cardiology residents; and one radiology fellow. The studies also diverged in terms of the field of medicine in which VR was applied. To date, there have been no systematic reviews of the topic involving a homogenous population. The details of the five studies evaluated are shown in Table 1.

**Table 1:** Details of the studies evaluated.

Study	Design	Participants	Procedure	Intervention	Comparator	Results	Kirkpatrick level of impact
Berry et al.^(10)^	Randomized clinical trial without questionnaire	12 novice endovascular surgeons	Iliac artery stenosis revascularization	Procedicus-VIST VRS training	Porcine training	Both methods were equivalent for endovascular skill acquisition, although the porcine training was more challenging	2B
Chaer et al.^(7)^	Randomized clinical trial with pre- and post-task questionnaire	20 general surgery residents	Catheter-based intervention	Procedicus-VIST VRS training, specific instruction, and didactic lectures	Specific instruction and didactic lectures, without simulation	Simulator training improved the performance of residents in the operating room	4B
Johnson et al.^(12)^	Randomized clinical trial with pre- and post-task questionnaire	14 interventional radiology residents	Seldinger technique	Seldinger VRS and traditional resident training	Traditional resident training	On average, participants who received VRS training overall performed significantly better	4B
Knudsen et al.^(20)^	Randomized clinical trial with pre- and post-task questionnaire	31 medical students, 31 residents, and 1 fellow	Percutaneous renal access puncture	PERC Mentor VRS training	Traditional resident training (no additional training)	Participants who received VRS training showed significant improvement in almost 80% of the parameters measured, whereas those in the control arm showed no significant improvement in any of the parameters	2B
Nesbitt et al.^(4)^	Comparative study with pre-task questionnaire	24 medical students with no prior endovascular experience	Left renal artery catheterization and confirmatory angiogram via right femoral artery access	ANGIO Mentor VRS training	PHCM	Both methods improved the learning of endovascular procedures, although the PHCM had a longer learning curve, with more gradual improvement	2B

In a comparative study involving 24 medical students with no previous endovascular training, Nesbitt et al.^(4)^ were able to establish the transferability of skills, showing that the learning curve was shorter when a VRS (ANGIO Mentor; Simbionix, Cleveland, OH, USA) was used in combination with a PHCM. The students were randomly and equally divided into two groups: those using the PHCM alone (control group); and those using the VRS and the PHCM. A pre-task questionnaire was applied, after which the students were familiarized with the operating instructions for the devices. The procedure chosen for evaluation was left renal artery catheterization, confirmed by fluoroscopy (angiogram). Videos of the procedures were then evaluated by two experienced vascular surgeons who were blinded to the methods used. Given that the mean number of attempts required to achieve technical success in a particular procedure ranges from two to seven, the authors established a limit of eight attempts per student, to avoid a learning bias. After that step had been completed, a crossover step with a single attempt was carried out in order to evaluate the applicability of using the different teaching methods. The authors concluded that the learning curve of the students who used the VRS reached a plateau after the second attempt, whereas the students in the control group had greater difficulty and consequently a longer learning curve. In the VRS group, the fluoroscopy times were shorter and smaller quantities of contrast medium were administered. The authors emphasized the importance of comparing different methods of medical teaching in endovascular procedures and recognized the need for additional studies to verify that such skills are transferable to real life.

Chaer et al.^(7)^ evaluated the effectiveness of using a simulator as a teaching method among general surgery residents with no prior endovascular experience. All of the participants were provided with reading material on basic catheter-based intervention for lower-extremity occlusive disease and attended a lecture on the topic. They were then randomly divided into two groups of 10 participants each. Those in one group received additional training in the technique on a simulator, whereas those in the other group did not. The result was evaluated by experienced surgeons who were blinded to the training status of the residents at the time of the surgery and used an 18-point checklist of the steps required to complete the procedure, including diagnostic angiography, angioplasty, and stenting. After two consecutive mentored interventions, the authors found that the group trained with the simulator received better scores for almost all aspects of the procedure, the difference in relation to the control group being even more pronounced for the second procedure. Overall, there were no significant differences between the two groups for the following aspects: advancing the femoral wire; mounting and advancing the catheter over the wire; and acquiring the image after percutaneous transluminal angioplasty. However, the participants trained with the simulator scored significantly higher for aspects such as knowledge of the anatomy, correct catheter positioning, balloon inflation, balloon pressure, and definition of the area of stenosis.

Employing porcine training, a teaching method that is lacking in robustness but is widely used in medicine, Berry et al.^(10)^ compared it with training on a VRS (Procedicus-VIST; Mentice, Gothenburg, Sweden). Given the space required, the ethical context, and the fact that healthy animals are used, the porcine training method does not have the high accuracy needed for the transfer of the skills learned to a sick patient. Twelve novice endovascular surgeons received two days of training for internal iliac artery revascularization with one or both of the methods, in different orders, prior to performing the procedure. Two experienced interventional radiologists, working independently, evaluated the videos of the procedures. The students who received VRS training had scores that were consistently and significantly higher than were those of the students who received porcine training. After the procedures, the participants completed a questionnaire designed to evaluate their opinions. Most agreed that both methods should be included in the academic context and that mentors are undoubtedly irreplaceable in surgical practice. It is also notable that when the students received the VRS training prior to the porcine training, the total score for both methods improved for the second procedure performed. However, when the porcine training was received first, the total score for the VRS method did not improve. The authors stated that the skills learned via VRS training may be transferable to the porcine model. The students who received VRS training had an advantage and acquired skills that were transferable, although few were able to finalize the iliac artery revascularization without a mentor, demonstrating that VRS training should be a complement to, rather than a substitute for, traditional teaching methods.

One of the puncture techniques most widely used in interventional radiology, known as the Seldinger technique, consists in making a single incision and inserting a coaxial system, which makes it possible to introduce and change the various materials, such as guidewires and catheters, that are used during interventional procedures. Johnson et al.^(12)^ evaluated methods of teaching the Seldinger technique in a randomized clinical trial conducted in three stages. With the aim of evaluating the learning of this technique by a VR-based method, 35 interventional radiology mentors carried out, on a VRS, the necessary steps to perform the procedure. In the second stage, the authors evaluated the performance of the experienced interventional radiologists in relation to the novices and concluded that those who already had technical skill scored higher in almost all aspects, especially regarding puncture time. In the final stage, designated “validation of the transfer of training”, the authors defined the applicability of this teaching method in real life. To that end, 14 interventional radiology residents were randomly divided into two groups: those receiving technical instructions with training on the VRS; and those receiving only verbal instructions (control group). The students who had previously trained on the VRS performed significantly better for all of the criteria than did those in the control group, indicating that the VR-based educational intervention had a direct impact.

In a study involving 63 participants (31 medical students, 31 residents, and one fellow), Knudsen et al.^(20)^ evaluated the applicability of VR to the teaching of percutaneous nephrostomy. The participants first underwent baseline testing on a VRS (PERC Mentor; Simbionix), after which they were randomized to receive either no additional training (control arm) or two 30-min sessions of training on the VRS (intervention arm). The participants in the intervention arm showed significant improvement in 11 of the 14 parameters measured, whereas those in the control arm showed no significant improvement in any of the parameters measured. The values for variables such as fluoroscopy time, puncture attempts, and vascular injuries were lower in the intervention arm, demonstrating a real shortening of the learning curve, as well as suggesting that VR-based teaching could reduce morbidity and mortality among patients undergoing percutaneous nephrostomy.

## DISCUSSION

Created in 1965 by Ivan Sutherland and initially tested in the field of computer science, VR has grown to cover a variety of scenarios. It has been available for use in interventional radiology since 1996, when the first interactive simulator was developed^(21)^. Since then, it has been shown to have numerous benefits and is constantly being transformed. However, it has some inherent limitations that should be mentioned^(22)^. Headaches and dizziness, as well as the cost and ergonomic limitations, continue to be the main challenges to be met in the use of the technology^(22)^.

Among the studies evaluated in this systematic review, there was heterogeneity of the populations evaluated and of the procedures included for analysis. The aim, therefore, our objective was to provide up-to-date information regarding the use and effectiveness of VR as an educational method in interventional radiology.

In all five of the studies analyzed, the authors concluded that VR-based teaching promotes the transfer of skills, the Kirkpatrick level of impact ranging from 2B to 4B. In three of the studies, the analysis referred mostly to the learning/performance of the students or residents as a direct result of the educational intervention (Kirkpatrick level 4B), whereas it referred to changes in the attitudes or perceptions of the participants in relation to the teaching and learning (evidence level 2B) in two. Among the analyses that were classified as Kirkpatrick level 2B, the control group was analyzed using other methods of teaching, such as porcine and cadaver training, which probably differ less significantly than do classes and seminars, because they involve dynamic learning.

Three of the studies evaluated involved basic interventional radiology techniques-catheterization, puncture, and the Seldinger technique, respectively. Another study evaluated percutaneous nephrostomy, and yet another assessed a slightly more complex procedure known as iliac artery stenosis revascularization. It should be stressed that the questions evaluated varied among the samples, although the main focus in all of the studies was the effectiveness of the teaching method. In relation to the population evaluated, residents and novice physicians were more often involved than were experienced surgeons, underscoring the fact that the former take greater advantage of VR-based training, thus shortening the learning curve.

Surgery is a medical specialty that changes constantly over the years^(22)^. Interventional radiology, which is one aspect of surgery, encompasses minimally invasive procedures, translating into lower cost, less morbidity, and shorter hospital stays^(12)^. The greatest difficulties found during the process of learning interventional radiology procedures involve spatial and cognitive proprioception, as well as motor difficulties in using the new equipment, which can have catastrophic consequences at the beginning of a career^(14,22)^.

With the aim of improving the education, as well as improving and enriching medical knowledge, new VR-based methods are currently being introduced, as are methods employing augmented reality or even hybrid forms of the two^(7)^. The traditional “see one, do one, teach one” method is quickly being supplanted by the concept of “learning before doing”, saving inexperienced doctors from committing potentially avoidable errors^(7,23)^. According to the British National Health Service, approximately 10% of hospitalized patients suffer from potentially avoidable injuries, most of which are attributable to inadequate medical training^(12)^. However, in a study involving experienced doctors, Willaert et al.^(24)^ evaluated the concept known as “warm-up” and found that when carried out with a simulator, the “warm-up” improves the surgical performance of the team, reduces medical errors, reduces surgical time/costs, and increases the self-confidence of the team members. This concept, although new in medical practice, is long-established in music and sports, with the aim of complementing the development of the professional^(24)^.

The term “practice makes perfect” reflects a scenario in which interventional radiologists need to perform a certain number of procedures in order to then execute them accurately and thoroughly^(25)^. Studies suggest that approximately 10,000 course hours are needed in order to acquire these procedural skills at an experienced level^(26)^. With the aim of achieving this level of training in resident physicians, the use of simulators has come to the forefront in the teaching of interventional radiology procedures^(26)^. When VR-based methods are added to the teaching protocol, the operator is able to learn a technique safely and effectively, which promotes patient well-being^(27)^. Reductions in procedure times and in the number of technical errors are the most desired effects of this new resource^(27)^.

In the United States, more than five million central venous catheters (CVCs) are used every year, and complication rates are 5-8% higher when such catheters are manipulated by professionals with little or no experience^(28)^. Medical errors that are considered foreseeable are responsible for 21,000 deaths annually in the country^(29)^. In a study conducted in 2006 at Northwestern University Feinberg School of Medicine^(30)^, residents completed a simulation-based course in CVCs before starting an intensive care unit internship. The authors found that, after one year, the CVC-related bloodstream infection rate fell from 4.2 to 0.42 cases for 100 patients. In addition to reductions in morbidity and mortality, that resulted in significant cost savings. According to those same authors, the focus on minimizing foreseeable complications and shortening hospital stays has been the motivation for studies aimed at identifying effective methods of medical training.

In an economic analysis of endovascular skills training, Berry et al.^(31)^ concluded that although the cost of a VRS can be prohibitive, simulation-based courses could result in a five-year savings of more than US$ 390,000 in comparison with the use of traditional porcine training. The authors estimated that, in comparison with traditional teaching methods, such as classes, books, and the use of animal models, the annual savings achieved with simulation-based training would be US$ 62,410. When taking into account spending on treatment for CVC-related infections and on hospitalization, Cohen et al.^(30)^ estimated an annual savings of approximately US$ 820,000, together with annual reductions of 137 fewer hospital admissions and approximately 120 fewer days in intensive care units. In addition, fluoroscopy time and the quantity of contrast medium used are two factors commonly found to have decreased in most of the randomized clinical trials evaluated in this review. The associated cost savings likely increase the overall savings attributed to the use of simulation-based training.

Institutions such as the European Board of Vascular Surgery and the German Society for Vascular Surgery and Vascular Medicine have already implemented or expressed an interest in including simulation-based training as a compulsory part of medical education^(14)^. However, it remains unknown which simulators are most effective for teaching, in which populations they should actually be implemented, and for what purposes they are indicated. Additional time will be needed in order to synthesize data that are more homogenous and objective, in order to perform a robust analysis. However, VR, as evaluated in the studies reviewed here, has a promising future, with numerous advantages in terms of skill acquisition and cost benefits, as well as reductions in patient morbidity and mortality. Incorporating this technology into medical school curricula will undoubtedly improve learning, as well as honing the technical skills of surgeons.

There is still a lack of studies involving homogenous target populations and specifically evaluating the application of the currently available simulators for teaching the various interventional radiology procedures. There have also been few studies evaluating VR-based training of experienced professionals.

## CONCLUSION

Our efficacy analysis of the studies selected in this systematic review revealed that the use of VR for training in interventional radiology resulted in the acquisition of transferable skills. Among the studies evaluated, there was a consensus that VR-based methods of teaching have many benefits. Although such methods can be used in conjunction with other methods of teaching interventional radiology, VR-based training should not be viewed as a substitute for traditional modes of training or for mentoring.

## References

[1] Silva AB, Amorim AC (2009). A Brazilian educational experiment: teleradiology on web TV. J Telemed Telecare.

[2] Duarte ML, Santos LR, Guimarães Júnior JB (2020). Learning anatomy by virtual reality and augmented reality. A scope review. Morphologie.

[3] Taylor DCM, Hamdy H (2013). Adult learning theories: implications for learning and teaching in medical education: AMEE Guide No. 83. Med Teach.

[4] Nesbitt CI, Tingle SJ, Williams R (2019). Educational impact of a pulsatile human cadaver circulation model for endovascular training. Eur J Vasc Endovasc Surg.

[5] Cates CU, Lönn L, Gallagher AG (2016). Prospective, randomised and blinded comparison of proficiency-based progression full-physics virtual reality simulator training versus invasive vascular experience for learning carotid artery angiography by very experienced operators. BMJ Stel.

[6] Kreiser K, Gehling K, Zimmer C (2019). Simulation in angiography - experiences from 5 years teaching, training, and research. Rofo.

[7] Chaer RA, Derubertis BG, Lin SC (2006). Simulation improves resident performance in catheter-based intervention: results of a randomized, controlled study. Ann Surg.

[8] Brigham TJ (2017). Reality check: basics of augmented, virtual, and mixed reality. Med Ref Serv Q.

[9] Barsom EZ, Graafland M, Schijven MP (2016). Systematic review on the effectiveness of augmented reality applications in medical training. Surg Endosc.

[10] Berry M, Lystig T, Beard J (2007). Porcine transfer study: virtual reality simulator training compared with porcine training in endovascular novices. Cardiovasc Intervent Radiol.

[11] Berry M, Reznick R, Lystig T (2008). The use of virtual reality for training in carotid artery stenting: a construct validation study. Acta Radiol.

[12] Johnson SJ, Guediri SM, Kilkenny C (2011). Development and validation of a virtual reality simulator: human factors input to interventional radiology training. Hum Factors.

[13] Samadbeik M, Yaaghobi D, Bastani P (2018). The applications of virtual reality technology in medical groups teaching. J Adv Med Educ Prof.

[14] Aeckersberg G, Gkremoutis A, Schmitz-Rixen T (2019). The relevance of low-fidelity virtual reality simulators compared with other learning methods in basic endovascular skills training. J Vasc Surg.

[15] Markovic J, Peyser C, Cavoores T (2012). Impact of endovascular simulator training on vascular surgery as a career choice in medical students. J Vasc Surg.

[16] Ouzzani M, Hammady H, Fedorowicz Z (2016). Rayyan-a web and mobile app for systematic reviews. Syst Rev.

[17] Higgins JP, Thomas J, Chandler J (2008). Cochrane handbook for systematic reviews of interventions.

[18] Buckley S, Coleman J, Davison I (2009). The educational effects of portfolios on undergraduate student learning: a Best Evidence Medical Education (BEME) systematic review. BEME Guide No. 11. Med Teach.

[19] Steinert Y, Mann K, Centeno A (2006). A systematic review of faculty development initiatives designed to improve teaching effectiveness in medical education: BEME Guide No. 8. Med Teach.

[20] Knudsen BE, Matsumoto ED, Chew BH (2006). A randomized, controlled, prospective study validating the acquisition of percutaneous renal collecting system access skills using a computer based hybrid virtual reality surgical simulator: phase I. J Urol.

[21] Uppot RN, Laguna B, McCarthy CJ (2019). Implementing virtual and augmented reality tools for radiology education and training, communication, and clinical care. Radiology.

[22] Gallagher AG, Smith CD (2003). Human-factors lessons learned from the minimally invasive surgery revolution. Semin Laparosc Surg.

[23] Mirza S, Athreya S (2018). Review of simulation training in interventional radiology. Acad Radiol.

[24] Willaert WIM, Aggarwal R, Daruwalla F (2012). Simulated procedure rehearsal is more effective than a preoperative generic warm-up for endovascular procedures. Ann Surg.

[25] Patel R, Dennick R (2017). Simulation based teaching in interventional radiology training: is it effective?. Clin Radiol..

[26] Gould D (2010). Using simulation for interventional radiology training. Br J Radiol.

[27] Aggarwal R, Black SA, Hance JR (2006). Virtual reality simulation training can improve inexperienced surgeons' endovascular skills. Eur J Vasc Endovasc Surg.

[28] Huang CY, Thomas JB, Alismail A (2018). The use of augmented reality glasses in central line simulation: "see one, simulate many, do one competently, and teach everyone". Adv Med Educ Pract..

[29] Wang CL, Chinnugounder S, Hippe DS (2017). Comparative effectiveness of hands-on versus computer simulation-based training for contrast media reactions and teamwork skills. J Am Coll Radiol..

[30] Cohen ER, Feinglass J, Barsuk JH (2010). Cost savings from reduced catheter-related bloodstream infection after simulation-based education for residents in a medical intensive care unit. Simul Healthc.

[31] Berry M, Hellström M, Göthlin J (2008). Endovascular training with animals versus virtual reality systems: an economic analysis. J Vasc Interv Radiol.

